# Saffron Crudes and Compounds Restrict MACC1-Dependent Cell Proliferation and Migration of Colorectal Cancer Cells

**DOI:** 10.3390/cells9081829

**Published:** 2020-08-03

**Authors:** Nazli Güllü, Dennis Kobelt, Hassan Brim, Shaman Rahman, Lena Timm, Janice Smith, Akbar Soleimani, Stefano Di Marco, Silvia Bisti, Hassan Ashktorab, Ulrike Stein

**Affiliations:** 1 Experimental and Clinical Research Center, Charité—Universitätsmedizin Berlin, and Max-Delbrück-Center for Molecular Medicine, Robert-Rössle-Straße 10, 13125 Berlin, Germany; nazli.guellue@mdc-berlin.de (N.G.); dennis.kobelt@mdc-berlin.de (D.K.); rahman.pharma87@googlemail.com (S.R.); Lena.Timm@epo-berlin.com (L.T.); j.smith@mdc-berlin.de (J.S.); 2 German Cancer Consortium (DKTK), Heidelberg, Im Neuenheimer Feld 280, 69120 Heidelberg, Germany; 3 College of Medicine & Cancer Center, Howard University 2041 Georgia Av. NW, Washington, DC 20059, USA; hooman1350@yahoo.com; 4 Center for Synaptic Neuroscience and Technology, The Italian Institute of Technology, IRCCS Ospedale Policlinico San Martino, 16132 Genova, Italy; Stefano.DiMarco@iit.it; 5 NetS3 Laboratory Neuroscience and Brain Technologies (NBT), The Italian Institute of Technology (IIT), Via Morego 30, 16128 Genova, Italy; Silvia.Bisti@iit.it; 6 Consorzio Interuniversitario INBB Istituto Nazionale Biostrutture e Biosistemi, V.le Medaglie D’Oro, 305, 00136 Roma, Italy

**Keywords:** MACC1, colorectal cancer, metastasis, DCLK1, natural compounds

## Abstract

The high mortality rate of colorectal cancer (CRC) patients is directly associated with metastatic dissemination. However, therapeutic options specifically for metastasis are still limited. We previously identified Metastasis-Associated in Colon Cancer 1 (MACC1) as a major causal metastasis-inducing gene. Numerous studies confirmed its value as a biomarker for metastasis risk. We investigated the inhibitory impact of saffron on MACC1-induced cancer cell growth and motility. Saffron crudes restricted the proliferation and migration of MACC1-expressing CRC cells in a concentration- and MACC1-dependent manner. Saffron delays cell cycle progression at G2/M-phase and does not induce apoptosis. Rescue experiments showed that these effects are reversible. Analysis of active saffron compounds elucidated that crocin was the main compound that reproduced total saffron crudes effects. We showed the interaction of MACC1 with the cancer stem cell (CSC) marker DCLK1, which contributes to metastasis formation in different tumor entities. Saffron extracts reduced DCLK1 with crocin being responsible for this reduction. Saffron’s anti-proliferative and anti-migratory effects in MACC1-expressing cells are mediated by crocin through DCLK1 down-regulation. This research is the first identification of saffron-based compounds restricting cancer cell proliferation and motility progression via the novel target MACC1.

## 1. Introduction

Colorectal cancer (CRC) is one of the most common cancers with metastasis formation responsible for the majority of cancer related deaths [1,2]. It has been estimated that 90% of the CRC-specific mortality were hinged on metastatic spread [3,4]. Despite treatment improvements, specific therapeutic options are limited [2]. Colorectal cancer can be classified into consensus molecular subtypes that are characterized by different molecular backgrounds including microsatellite stability, CpG island methylation, mutational status, immune infiltration or signaling pathway activity (e.g., WNT/β-catenin) [5]. Profound knowledge of the molecular background of a tumor is needed for successful treatment. Here, causal biomarkers, either as single genes or as member of a molecular signature, driving tumor development, progression and metastasis formation, play an important role.

We identified Metastasis-Associated in Colon Cancer 1 (MACC1) as a causal metastasis-promoting oncogene [6]. Elevated expression of this gene increases proliferation, migration, and invasiveness of cancer cells. MACC1 promotes epithelial-mesenchymal transition (EMT), a process changing an epithelial mostly low motile phenotype of cells towards a mesenchymal phenotype that is often linked to an increased motility. Further, it increases angiogenesis and cancer stemness [6,7]. Clinically it was shown that MACC1 expression promotes tumor progression and metastasis formation in more than 20 different solid tumor entities, including CRC [6,7]. High MACC1 expression is significantly linked with decreased 5-year metastasis-free and overall survival, establishing its role as prognostic biomarker for CRC [6,7]. The predictive value of MACC1 was shown e.g., for CRC and breast cancer [8,9]. These studies highlight MACC1 as a target for cancer therapy.

Many research groups tried to improve existing treatment regimens and develop new drugs to gain the desired remedial effects targeting malignant tumors. Insufficient outcomes and undesirable side effects of these drugs urged researchers to study new approaches to achieve better treatment outcomes. Many studies attempt to improve treatment by combining approved drugs with natural compounds and to establish natural compound-based treatment regimens [10]. One of these promising natural products is saffron (Crocus sativus). Therapeutic effects of saffron have been known since ancient Egyptian time [11]. Saffron contains three major compounds responsible for its activity: crocetin, safranal and crocin. The effects of saffron and its compounds have been investigated by different research groups on several cancer entities [12,13]. These in vitro and in vivo studies revealed anti-cancer effects via numerous mechanisms, including inhibition of DNA and RNA synthesis, activation of STAT3, modulation of the immune system, suppression of telomerase activity, inhibition of cell proliferation, modulation of metabolism, inhibition of cell cycle progression, and stimulation of cell-to-cell gap junction [14,15,16,17,18]. Further, recent studies suggested possible anti-metastatic effects of this natural product, including downregulation of matrix metalloproteinases (MMP) -2, -9, N-cadherin expression reduction, and increase of E-cadherin [18,19,20,21,22].

Interestingly, besides other effects, saffron is also able to target cancer stem cells (CSC) via the decrease of DCLK1 [23]. This population of cells is important for tumor (re)constitution and metastasis formation [23].

The entire remedial effect of saffron and its compounds on tumor progression is not entirely understood. In this study, we have investigated the impact of saffron crudes and selected saffron compounds on MACC1-induced phenotypes. This is the first identification of saffron-based compounds restricting cancer cell proliferation and motility via the novel target MACC1.

## 2. Methods

### 2.1. Cell Cultures

The cell lines SW480 and SW620 were obtained from the American Type Culture Collection (Manassas, VA, USA). The cells were maintained in RPMI and DMEM (Invitrogen, Darmstadt, Germany), respectively, supplemented with 10% (*v*/*v*) fetal calf serum (FCS) (Bio & Sell, Feucht, Germany) at 37 °C with 5% CO_2_ in a humidified atmosphere. MACC1 was knocked-out in SW620 by Crispr-Cas9: SW620/KO-MACC1 (control: SW620/Control). SW480/MACC1 cells were generated by stable transfection of pcDNA3/MACC1 (controls: SW480/Control) [6] (Supplementary Figure S1). HEK293T cells were cultured in DMEM/10% FCS. They were transduced with lentiviral DCLK1-GFP and MACC1-RFP for the MACC1-DCLK1 interaction studies. Viruses were produced following standard procedures using the plasmids psPAX2 (Addgene 12260), pMD2.G (Addgene 12259) and pLenti vectors (Origene, Rockville, MD, USA) coding for GFP-tagged DCLK1 and RFP-tagged MACC1. DCLK1 cDNA was a kind gift of C. W. Houchen (University of Oklahoma Health Sciences Center, Oklahoma City, OK, USA). Positive cells were selected by FACS.

### 2.2. Preparation of Saffron Crudes, Compounds and DCLK1 Inhibitors

Experiments were performed with four different saffron crudes obtained from Gulf Pearls, SPRL (Brussels, Belgium). For each experiment, fresh, sterile filtered saffron crudes (20 mg saffron crude per ml water, 24 h shaking at room temperature) were prepared. Saffron crudes were used at final concentrations of 1%, 2.5%, 5%, and 10% in complete growth medium. The difference between the saffron samples is the concentration of the main compounds such as crocin and safranal. A final compound concentration of 5 mM safranal, 5 mM crocin, and 5 µM crocetin has been used. DCLK1 inhibitor LRRK2-IN-1 has been used in concentrations between 0.78–25 µM.

### 2.3. RNA Extraction and Quantitative PCR

For the isolation of total RNA, the GeneMatrix Universal RNA purification Kit (EURx, Gdansk, Poland) has been used according to the manufacturer’s instructions. Isolated RNA was eluted with ultrapure water. RNA concentration was measured using a NanoDrop 2000 spectrophotometer (Peqlab, Erlangen, Germany). For each sample, 50 ng total RNA has been reverse transcribed (Biozym, Hessisch Oldendorf, Germany). The cDNAs were amplified using SYBR green chemistry (Biozym) according to the manufacturer’s instructions using the LightCycler 480 (Roche Life Science, Mannheim, Germany) with the primer sequences listed in Supplementary Table S1.

### 2.4. Co-Immunoprecipitation (Co-IP)

For Co-IP experiments, cells were scratched off with 500 µL Protease-Inhibitor containing IP-lysis buffer (Roche Diagnostics, Risch, Switzerland). Cell lysates were centrifuged for 45 min at 14,000 rpm at 4 °C. Proteins were pulled-down with 2 µg of the respective antibody overnight at 4 °C. Then 20 µL of G-agarose beads were added (Alpha Diagnostic International Inc., San Antonio, TX, USA) and incubated for 4 h at 4 °C on a rotational shaker. After washing with lysis buffer, we eluted the protein-complexes with DTT (AppliChem GmbH, Darmstadt, Germany) containing LDS-buffer (Thermo Fisher Scientific, Waltham, MA, USA). Eventually, we spun at 2500 rpm for 5 min at 4 °C, and 20 µL of supernatant were transferred to the SDS page.

### 2.5. Protein Extraction and Western Blot

Saffron treated cells were lysed in RIPA buffer. Protein concentrations were quantified using the Pierce BCA Protein Assay (Thermo Fischer Scientific) according to manufacturer’s instructions. For MACC1 and DCLK1 30 μg, cleaved-caspase-3 and cleaved-PARP 60 μg protein lysates were separated using 12% sodium dodecyl sulfate polyacrylamide gels. The proteins were transferred to nitrocellulose membranes and incubated with the respective primary antibody followed by HRP-labelled secondary antibodies. Bound antibodies were detected by incubating the membranes in a chemiluminescent substrate (Advansta, San Jose, CA, USA). Protein bands were visualized by exposure to SuperRX X-ray films (Fujifilm, Tokyo, Japan). β-actin and vinculin were used as loading controls.

### 2.6. MTT Assay

Cells were treated with each of the four different saffron crudes for 24 h in 96-well plates. After incubation with MTT (Thermo Fischer Scientific), cells were dissolved in DMSO, and absorbance was measured with a multi-well plate reader (InfiniteM200 PRO, Tecan, Männedorf, Switzerland).

### 2.7. Flow Cytometry

To assess the cell cycle state, cells were treated with saffron extracts for 24 h, then harvested, washed, and fixed with 66% ethanol for 2 h, at 4 °C. Next, cells were pelleted and resuspended in 200 μL RNAse and PI containing staining solution (20 min, 37 °C). The samples were analyzed using LSR 2 flow cytometry (BD Bioscience, San Jose, CA, USA).

### 2.8. Migration Assay

For analyzing cell migration, the Boyden Chamber (Corning, New York, NY, USA) assay has been used. Serum starved (6 h 0.5% FCS) cells were seeded in the upper chamber with 0.5% FCS; 10% FCS served as chemoattractant in the lower chamber. Saffron crudes and crocin were added to both chambers. Migrated cells in the lower chamber were collected after 16 h and lysed with 20 µL of CellTiter-Glo reagent (Promega, Madison, WI, USA). Luminescence was measured with a multi-well plate reader (Infinite M200 PRO).

### 2.9. Live Cell Imaging

To detect the effect of saffron extracts on cell proliferation, IncuCyte ZOOM system (Essen Bioscience, MI, USA) has been used. 1 × 10^4^ cells/well were seeded in 96-well plates and treated with different concentrations of saffron extracts or its compounds. Cell proliferation was assessed every 2 h and analyzed using the integrated software.

To rescue saffron effects, cells were exposed to the saffron extracts for 24 h with subsequent exposure to saffron-free culture medium.

### 2.10. Kinase Assay

For the kinase assay, DCLK1 was isolated from HEK293T cells after lentiviral transduction. To isolate the protein, Nanotrap-based (ChromoTek, Martinsried, Germany) Co-IP for GFP was performed following manufacturers recommendations. Cleared lysates of 10^7^ cells were incubated with Nanotrap-coupled beads for 1 h. After washing, the protein-loaded beads were directly used for the kinase assay. Five-hundred nanograms of recombinant MACC1 (Origene, Rockville, MD, USA) was incubated at 37 °C with 10 µg isolated DCLK1 in kinase buffer containing 0.3 µM γP^32^ ATP (Perkin Elmer, Rodgau, Germany), 80 mM HEPES pH 7.5, 4 mM MgCl_2_, 4 mM MnCl_2_, 1.6 mM DTT, and 70 µg/mL PEG_20,000_ for 15, 30, 45, and 60 min. Samples were heat-inactivated and separated on 10% polyacrylamide gels. The gels were dried on Whatman paper and bands were visualized with imaging plates (BAS-IP MS 2340, Fujifilm, Japan) using an Amersham Typhoon FLA 7000 (GE Healthcare, Freiburg, Germany).

### 2.11. FRET Measurement

For Förster resonance energy transfer (FRET) measurements, GFP- and RFP-tagged proteins were overexpressed by lentiviral gene transfer in HEK293T cells. FACS-selected cells were seeded in rat collagen (Santa Cruz, Dallas, TX, USA) coated chamber slides (Ibidi, Gräfelfing, Germany). After 24 h, cells were fixed with 4% paraformaldehyde. FRET was analyzed at 63,000-fold magnification. Multiple consecutive images for GFP- and RFP-positive cells were acquired using corresponding filter channels. The pictures were analyzed in Image J (NIH, Bethesda, MD, USA) plugin ’FRETTY’. FRET efficacy has been utilized between 0 and 1.

### 2.12. Liquid Chromatography

Saffron samples have been characterized using HPLC and spectroscopy methods. Aqueous extracts were prepared according to the ISO 3632 standard. Ultraviolet–visible spectral data in the wavelength range of 200–600 nm and the intensities of the chromatographic peaks attributed to the saffron compound crocin was recorded [24].

### 2.13. Statistical Analysis

All statistics were performed with GraphPad Prism version 6.0 (La Jolla, CA, USA). Comparisons of two different groups were performed by Student’s t-test. For analysis of three or more groups, one-way analysis of variance (ANOVA) with Dunnet´s post-hoc test was employed. *p* values smaller than 0.05 were considered statistically significant (* = *p* < 0.05, ** = *p* < 0.01).

## 3. Results

### 3.1. Saffron Restricts Proliferation and Viability of CRC Cells with High MACC1 Expression

**Figure 1:**
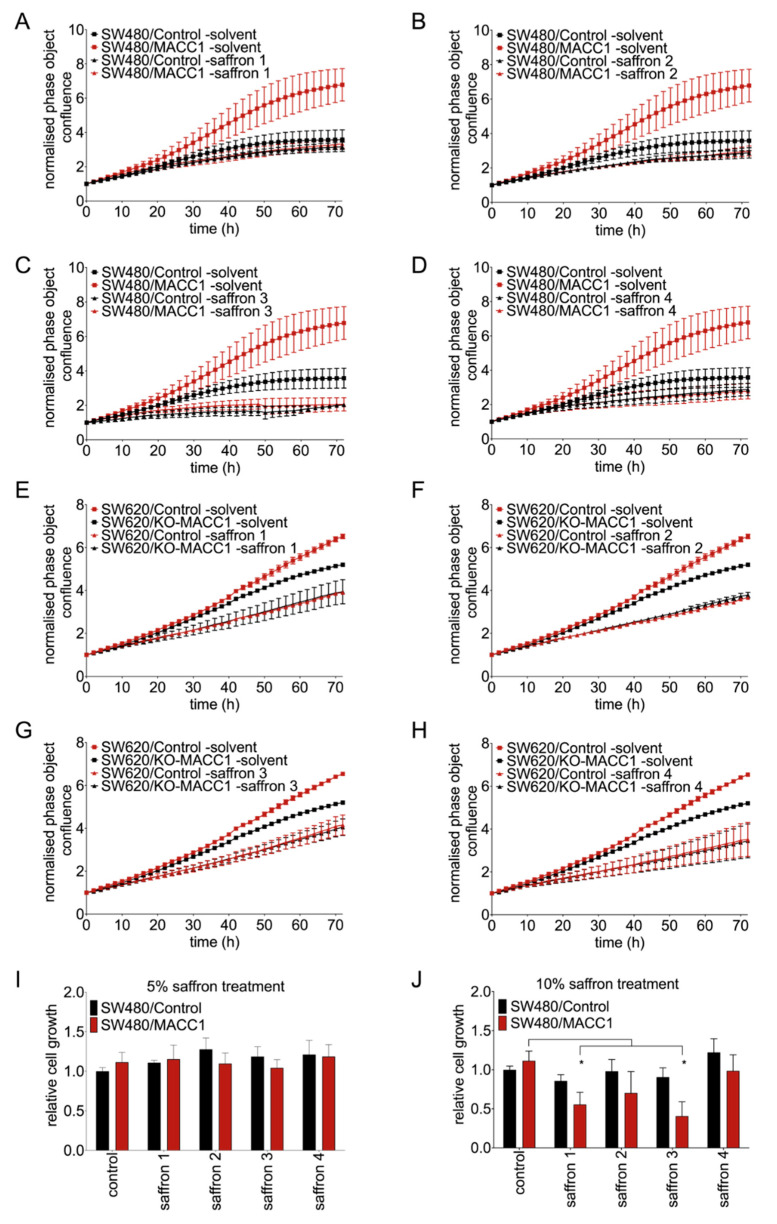
Saffron crudes reduced the proliferation and viability of CRC cells in a MACC1-dependent manner. CRC cells were treated with four different saffron crudes. Every second hour the confluence of the cells was measured by a live cell imaging system. Each saffron crude reduced the proliferation of both SW480/MACC1 (**A**–**D**) and SW620/Control cells (**E**–**H**). Cell viability was further assessed using MTT assay, and correlatively to the previous results, each saffron crude decreased the cell viability in a concentration-dependent manner. Cells treated with 5% saffron did not show a reduced viability (**I**) whereas the 2-fold increased amount of saffron reduced the cell viability most pronounced for saffron 1, 2 and 3 (**J**). Data are represented as mean ± SEM (*n* ≥ 3). *p* values were calculated using one-way ANOVA, * *p* < 0.05.

Initially, we evaluated the MACC1-dependent impact of four whole saffron crudes on cell proliferation. Saffron samples (saffron 1–4) of different origin were used in this study to generalize our findings for different saffron sources. We employed the MACC1 low-expressing CRC cell line SW480 and clones thereof with stable ectopic MACC1 overexpression (SW480/MACC1). We treated the cells with different saffron crudes for 72 h, and cell growth was monitored label-free using the IncuCyte live cell imaging system every 2 h. High MACC1-expressing cells without any treatment showed higher proliferation rates compared to the low MACC1-expressing cells. However, all MACC1 expressing cell lines treated with saffron crudes showed a decrease in proliferation. The reduction differed between the saffron samples. Three days after treatment with 10% of each saffron crude, MACC1-overexpressing SW480 cells showed a decrease in proliferation by 50%, 56%, 70%, and 59%, with saffron samples 1, 2, 3, and 4, respectively. The cell line SW480/control is affected by saffron treatment too, pointing to additional MACC1-independent effects. Lower concentration of saffron treatment did not lead to the same effect (Figure 1A–D, Supplementary Figure S2). To further confirm these results, similar experiments were performed in the SW620 cell line, which has an endogenously high MACC1 level. In this cell line, MACC1 was knocked out by CRISPR/Cas9. Similar effects were observed using saffron 1, 2, 3, and 4 crudes. They reduced the proliferation by 40%, 43%, 38%, and 47%, respectively (Figure 1E–H). The effect of saffron on SW620/KO-MACC1 cells was not as prominent as SW620/Control cells. This shows that the observed effect is mediated partially by MACC1, but points to additional effects not mediated via this molecule. To further examine the effect of different saffron crudes on the growth of the CRC cell lines, we treated the cells with different saffron crudes for 24 h and assessed the amount of living cells by MTT. Saffron crudes reduced the viability of SW480/MACC1 cells compared to solvent-treated cells in a concentration-dependent manner (Figure 1J). Each saffron sample reduced proliferation and the viability of the CRC cells with high MACC1 expression. However, due to different main compound ratios between different saffron samples, reduction rates differ. The strongest effects were observed at the highest concentration of 10% with saffron sample 3. Cell viability of SW480/MACC1 cells was reduced by 63% compared to control. A similar but less pronounced effect was observed with saffron samples 1, 2, and 4. The reductions were 49%, 36% and 11%, respectively. Using lower concentrations (5% saffron crude and below) of all tested saffron crudes showed no effects on cell viability (Figure 1I) and proliferation (Supplementary Figure S2).

### 3.2. Saffron Reduces the Migration Rate of MACC1-Expressing CRC Cell Lines

**Figure 2:**
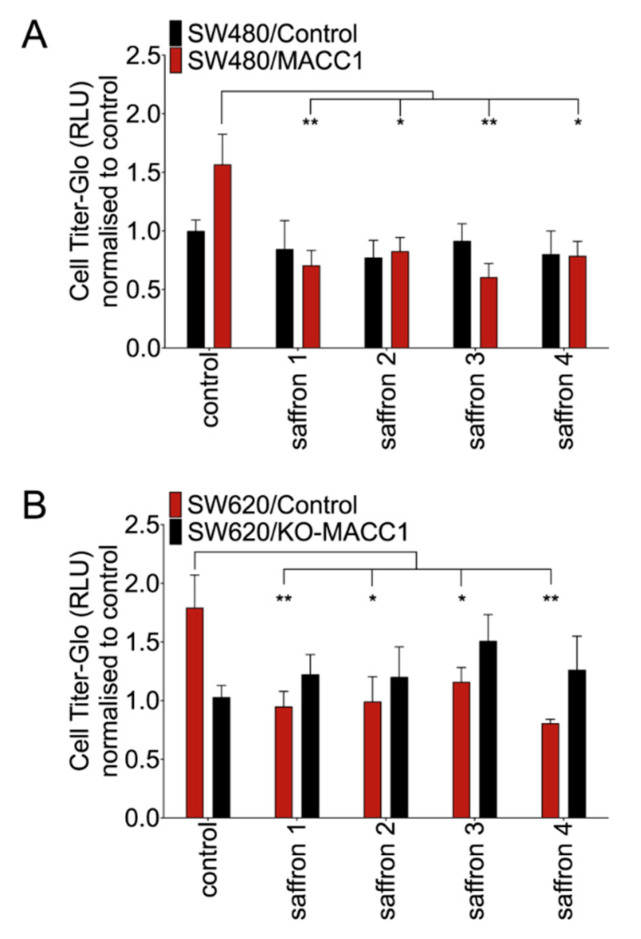
Saffron treatment reduced the migration of CRC cell lines. The low and high MACC1-expressing cells were treated with saffron crudes, and their migration capacity has been measured using the Boyden chamber assay. The values were normalized to control cells treated with solvent. Saffron crudes decreased the migratory capacity of the CRC cells in a MACC1-dependent manner in both SW480 (**A**) and SW620 (**B**) cell lines. Data are represented as mean ± SEM (*n* ≥ 3). *p* values were calculated using one-way ANOVA. * *p* < 0.05, ** *p* < 0.01**.**

To explore the effect of saffron crudes at the functional level, we assessed their impact on the motility of different CRC cell lines. The migration reduction in high MACC1-expressing CRC cells was observed after treatment with each of the saffron crudes. However, the reduction rates differed among saffron samples. The highest decrease (61%) of the migration has been observed with saffron 3 in SW480/MACC1 compared to solvent-treated cells. Saffron 1, 2 and 4 decreased migration by 55%, 47% and 50%, respectively (Figure 2A). This observation was further substantiated in SW620 cells. In the same direction, saffron 1, 2, 3, and 4 reduced migration of the SW620/Control cells by 46%, 42%, 33%, and 53%, respectively. None of the saffron crudes led to a significant migration decrease in SW620/KO-MACC1 cells (Figure 2B).

### 3.3. Saffron Restricts Proliferation of CRC Cells with High MACC1 Expression through Cell Cycle Arrest

To test if reduced proliferation is due to cell death, we performed rescue experiments. To elucidate if the effect of saffron on proliferation is reversible, we replaced saffron-containing medium after 24 h with fresh saffron-free culture medium.

**Figure 3:**
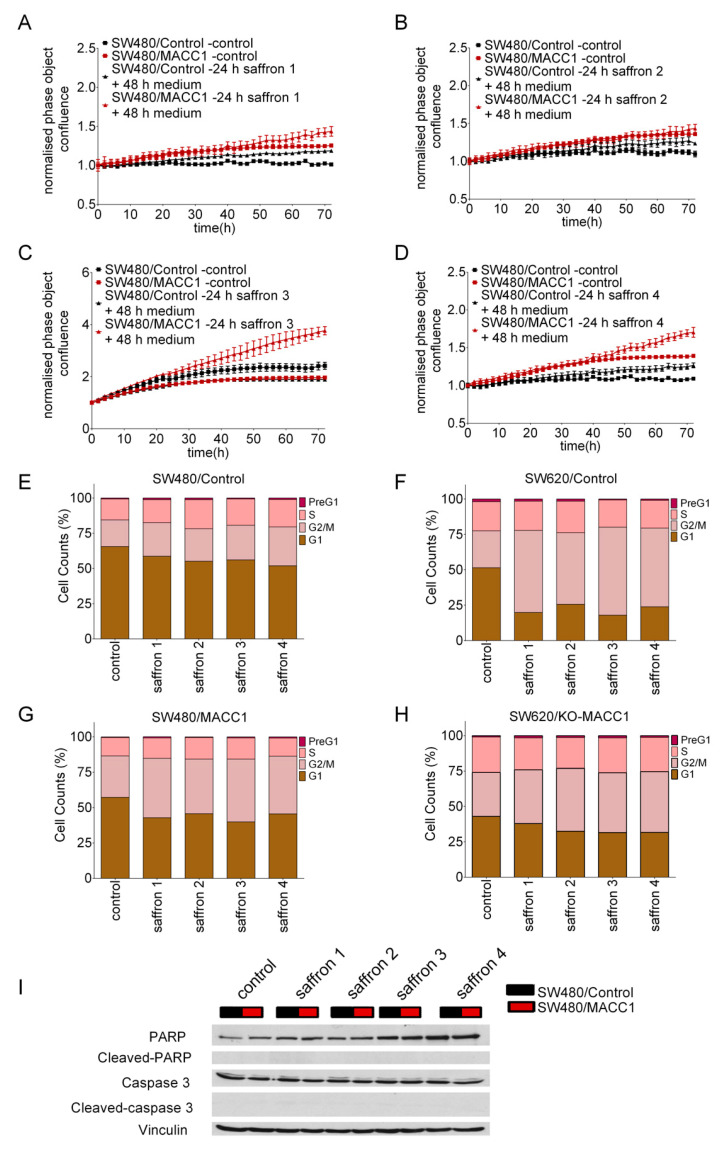
Saffron treatment led to cell cycle arrest upon MACC1 expression. Replacement of saffron-medium with saffron-free medium after 24 h increased the proliferation of CRC cells with high MACC1 expression. As a control, the cells were treated with freshly prepared saffron dilutions after 24 h (**A**–**D**). In agreement with the rescue experiment, via PI staining the cell cycle distribution of the cells was investigated. Twenty-four hour saffron exposure increased the cell portion at G2/M phase in MACC1 overexpressing CRC cells (**E**–**H**). Forty-eight hours of saffron treatment did not increase the cleaved-caspase 3 and cleaved-PARP level of the treated cancer cells compared to untreated controls (**I**).

High MACC1-expressing cells pre-treated with saffron showed an enhanced proliferation rate upon medium replacement (Figure 3A–D). This was most pronounced for saffron 3 (Figure 3C). Unexpectedly, fresh medium replacement after saffron 2 treatment did not lead to a proliferation rate enhancement (Figure 3B). To reveal how saffron leads to this effect, cell cycle arrest was investigated. Twenty-four hours of saffron 1, 2, 3, and 4 treatment increased the SW480/MACC1 cell portion at G2/M phase up to 43%, 32%, 51%, and 40% compared to the solvent-treated cells, respectively. More robust effects of the saffron treatment were observed in SW620 cell line. The cell fraction of MACC1-expressing positive cells at G2/M phase increased under saffron 1, 2, 3, and 4 treatments up to 58%, 50%, 62%, and 55% compared to the untreated controls, respectively. This effect was reduced in SW620/KO-MACC1 with no MACC1 expression. (Figure 3E–H). The MACC1 negative cell lines (SW480/control and SW620/KO-MACC1) are still affected by saffron treatment, pointing to additional MACC1-independent effects.

In different studies, it has been suggested that saffron induces apoptosis [25]. Therefore, we investigated cleaved-caspase-3 and PARP levels in CRC cells after saffron treatment. We did not detect any changes in the protein level of cleaved-caspase-3 or PARP (Figure 3I) under any saffron treatment.

### 3.4. Crocin Is the Main Compound Responsible for MACC1-Dependent Anti-Proliferative Effect of Saffron

**Figure 4:**
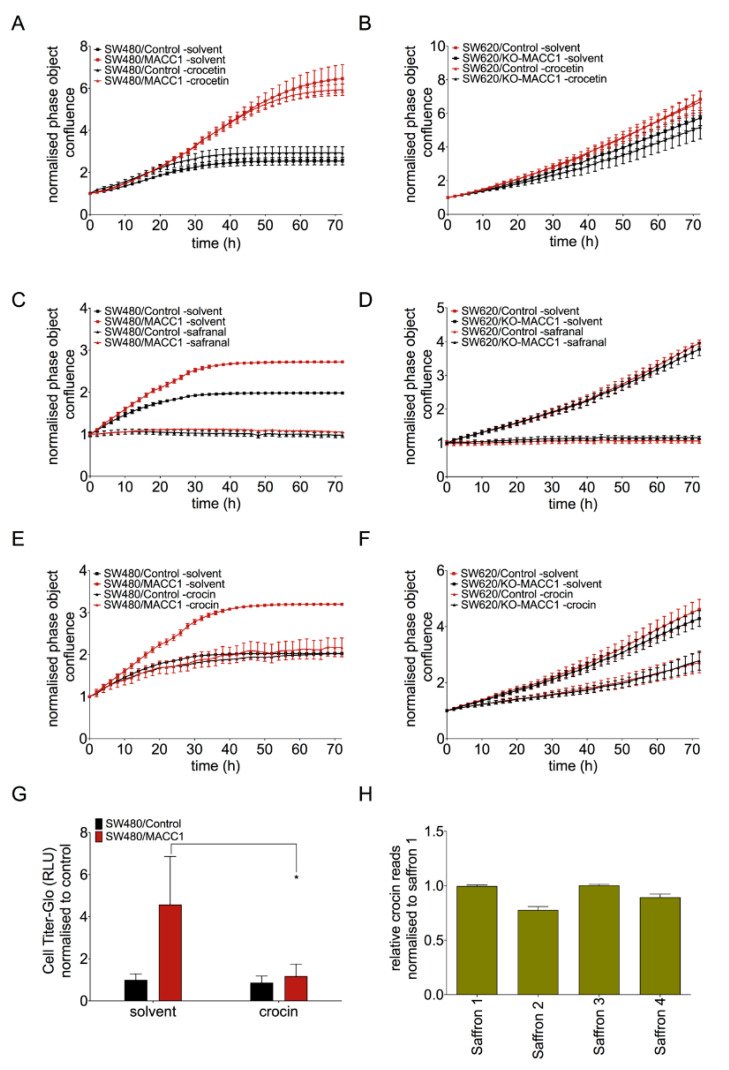
Crocin reproduced the MACC1-dependent effect of saffron. Cells were treated with different crocetin concentrations. Crocetin did not reduce the proliferation of both MACC1 high and low expressing cells (**A**,**B**). Safranal reduced the proliferation rate of both cell lines in a MACC1-independent manner (**C**,**D**). Crocin decreased the proliferation rate of the CRC cells with high MACC1 level (**E**,**F**). In addition, migration reduction in high MACC1-expressing CRC cells was observed under crocin treatment in Boyden chamber assay (**G**). Following this, via chromatography analysis, the direct read of crocin extract of each saffron was established. High variation of crocin has been observed between the four different saffron extracts (**H**). Data are represented as mean ± SEM (*n* ≥ 3). *p* values were calculated using Student’s *t*-test. * *p* < 0.05.

Major saffron compounds are crocetin, safranal and crocin [26]. It is not clear which compound mediates the MACC1-dependent effects. Therefore, we investigated the effect of these major compounds on CRC cell proliferation (Figure 4A–F). Crocetin treatment of CRC cells for 72 h did not lead to any proliferation reduction regardless of the MACC1 expression level (Figure 4A,B). Safranal reduced the proliferation of CRC cells independent of MACC1 level (Figure 4C,D).

However, crocin-treated SW480/MACC1 cells (high MACC1 expression) showed a 2-fold decreased proliferation compared to control (Figure 4E). In the SW480/Control cells (low MACC1 expression), the proliferation reduction was not observed upon crocin treatment. The same effect has been confirmed in SW620 cells (Figure 4F). The SW620/Control cells (high MACC1 expression) showed a 2-fold lower proliferation under crocin treatment compared to SW620/KO-MACC1 cells (no MACC1 expression) (Figure 4F). In addition, crocin treatment led to the reduction of the migration rate of SW480 cells with high MACC1 expression (Figure 4G).

In an independent liquid chromatography, the distribution of crocin in each saffron sample was analyzed. We observed a high variability among the different saffron samples in the crocin levels. While extracts of saffron 1 and 3 had the highest crocin amounts, saffron 2 and 4 showed lower amounts (Figure 4H).

### 3.5. Saffron Reduces DCLK1 Levels in CRC Cells

**Figure 5:**
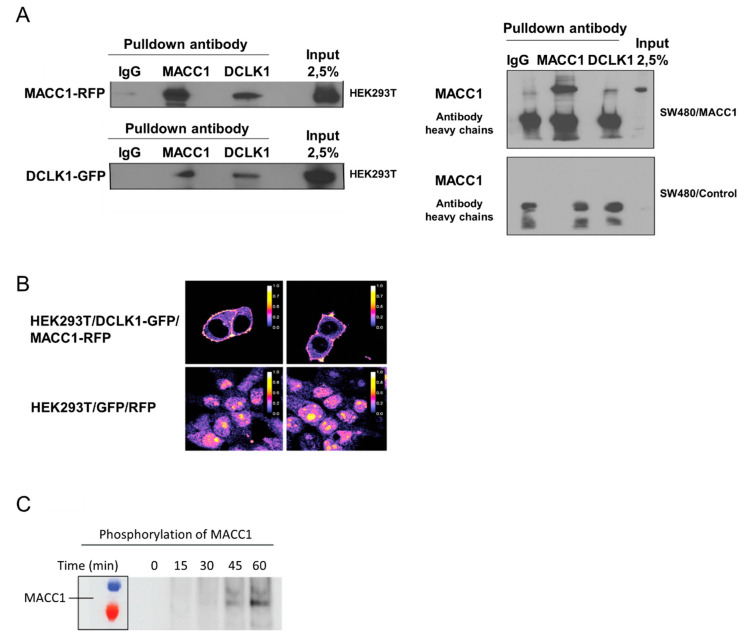
DCLK1 interacts with MACC1, leading to the phosphorylation of MACC1. MACC1-DCLK1 interaction is shown by Co-IP (**A**). Using FRET the interaction was analyzed in living cells. As a color scale, Firescala was used and the FRET efficacy was utilized between 0 and 1. While 1 indicates high FRET efficacy, 0 is the lowest. Highest FRET efficacy was detected at the cell membrane, which indicated the MACC1/DCLK1 interaction (**B**). MACC1 phosphorylation by DCLK1 is shown in a kinase activity assay with radiolabeled ATP. The samples were analyzed by immunoblotting and phosphorylated MACC1 was detected by autoradiography. The intensities of the phosphorylated MACC1 band were analyzed by Image J. The maximum phosphorylated MACC1 band at 60 min was defined as 100% of intensity (**C**).

Crocetin, a saffron compound, can reduce DCLK1 levels in vitro [23]. Therefore, we tested the molecular connection of MACC1 and DCLK1. We overexpressed MACC1 and DCLK1 fused to fluorescent markers in HEK293T cells. After pull-down of either protein, we found the other one as interaction partner. This interaction was confirmed in a CRC cell line with ectopic MACC1 overexpression (Figure 5A). DCLK1 is a transmembrane protein, whereas MACC1 is mainly located in the cytoplasm. Analyzing the location of DCLK1 and MACC1 in cells by FRET revealed that both proteins are interacting at the membrane (Figure 5B). To prove the productive nature of the interaction at the membrane, we used isolated recombinant proteins in a radioactive kinase assay. Incubating DCLK1 with MACC1, we found a time-dependent transfer of ^32^P to MACC1. This shows that both proteins are not only co-localized at the membrane, but that DCLK1 can phosphorylate MACC1 (Figure 5C).

**Figure 6:**
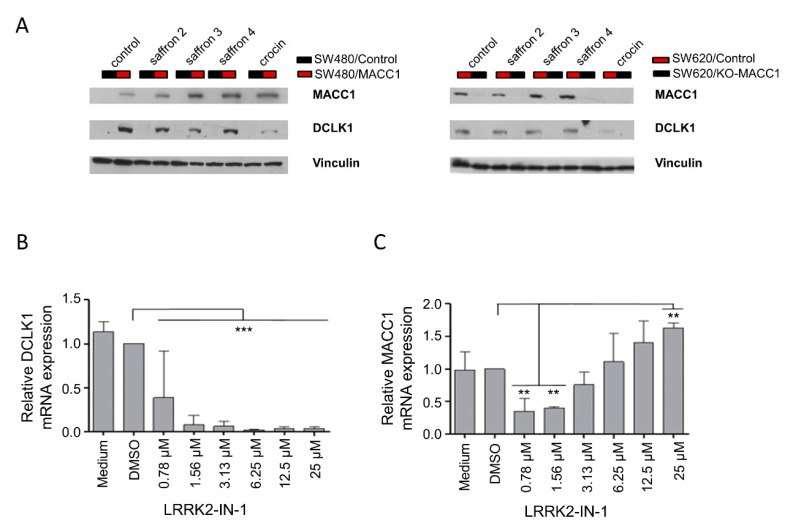
Saffron crudes reduced DCLK1 levels in CRC cells. Shown here are the MACC1 and DCLK1 levels in CRC cells after 48 h saffron 2–4 and crocin treatment. DCLK1 levels decreased upon 48 h saffron treatment; on the other hand, MACC1 levels increased under the same treatment (**A**). The DCLK1 inhibitor LRRK2-IN-1 led to the reduction of DCLK1 (**B**) and caused MACC1 expression change in a concentration-dependent manner (**C**). ** *p* < 0.01, *** *p* < 0.001.

DCLK1 has been discussed in several studies as a target for new therapeutic approaches in CRC [27,28,29]. In respect to this, we investigated the effect of saffron and crocin treatments on the DCLK1 expression levels. CRC cells with high MACC1 expression showed elevated levels of DCLK1 compared to the respective cell line with low MACC1 expression (Figure 6A, each control treated cell pair). Treatments with saffron crudes or crocin for 48 h reduced DCLK1 protein levels in high MACC1-expressing cells (Figure 6A).

Correlatively to this, we also studied the effect of the DCLK1 inhibitor, LRRK2-IN-1, which is an ATP competitive substance, on the DCLK1 and MACC1 expression of these cells. The DCLK1 inhibitor decreased the DCLK1 mRNA expression (Figure 6B). Additionally, MACC1 mRNA expression was decreased at low inhibitor doses (Figure 6C). At elevated inhibitor doses starting at 3.13 µM it also led to an induction of MACC1 mRNA expression in a concentration-dependent manner (Figure 6C).

Here we showed the ability of different natural saffron crudes and its crocin compound to inhibit MACC1-induced proliferation and motility, thereby reducing CRC metastatic potential. Mechanistically, we newly identified the interaction and direct phosphorylation of MACC1 by DCLK1, which is inhibited by saffron through downregulation of DCLK1. Therefore, the MACC1-DCLK1 interaction could be used as a therapeutic target for saffron extracts to inhibit metastasis formation mediated by MACC1.

## 4. Discussion

CRC is one of the most common cancers and causes of morbidity and mortality in the world [2,30]. Despite therapeutic developments, the 5-year overall survival rate of patients with metastasis is still low [31]. MACC1 is a metastasis driving gene, whose upregulation drops the 5-year metastasis-free survival rate of CRC patients to 15% compared to 80% for patients with low MACC1 expression [6,8]. MACC1 is involved in the regulation of cell viability, proliferation and migration. This is mediated through dysregulation of PI3K/AKT, MAPK/ERK and other pathways as reported by various research groups for different tumor entities [6,32]. Therefore, it is crucial to intervene in the carcinogenic, tumor- and metastasis-promoting effects of this oncogene. In the present study, we investigated the impact of the natural product saffron on the tumor-promoting impact of MACC1. We showed for the first time the concentration- and MACC1-dependent anti-proliferative and anti-migratory effects of saffron on CRC cell lines via the downregulation of the CSC marker DCLK1 (Graphical abstract).

In our study, we have chosen saffron, one of the promising natural products, which has been used for centuries to treat different diseases [33]. In recent years, the anti-tumor effects of this herb have been investigated by a considerable number of different research groups in different tumor entities, including lung, breast, and colon cancer [13]. These studies gave hints for the possible anti-metastatic effects of saffron crudes and its compounds by downregulating metastasis-associated genes such as MMP-2, -9 and Wnt pathway target genes including FZD7, NEDD9, VIM, and VEGF-α [19,34,35].

In the present study, we demonstrated that saffron crudes and its main compound crocin can be used to supplement current treatments for CRC. Indeed, total crudes from different saffron samples restricted proliferation of CRC cells with endogenous high MACC1 expression (SW620/Control) and with forced overexpression of MACC1 (SW480/MACC1) in a dose-dependent manner. The strongest effects were observed at the highest saffron extract concentration. When using lower saffron crude concentrations, only minor or no effects were observed.

There is a considerable amount of studies indicating that the ratio of compounds provided by saffron changes in different sources. Previous studies of different groups elucidated that the seed origin, the planting conditions like soil type, altitude, climate, and harvesting time affect the compounds composition of this herb [36,37,38]. Concordantly, in our study we observed that each of the saffron crudes led to different reduction rates of cell proliferation and motility. This likely reflects different compositions and concentrations of their active compounds. This is indeed the reason why typing and cataloging of different samples is needed to maintain preventive and therapeutic characteristics of saffron. In this work, the MACC1-dependent efficacy of saffron was analyzed in SW480 cells and CRISPR-Cas9-mediated MACC1 knock-out clones of SW620, both with very low MACC1 expression in comparison to MACC1-expressing counterparts. In both cell systems the saffron crudes did not inhibit motility by SW620/KO-MACC1 and SW480/Control due to the missing target (MACC1).

Previous studies suggested that saffron can induce apoptosis and cell cycle arrest in different tumor entities. Therefore, we further tested which pathway saffron affects when it downregulates MACC1. Using PI staining, we established that the tested saffron crudes induced the cell portion at the G2/M phase. In SW480/MACC1 cells, up to 45% of the cells were stalled in G2/M compared to the solvent-treated cells. Stronger effects of the saffron treatment were observed in SW620/Control cells with even higher MACC1 expression compared to SW480/MACC1. This result shows that saffron increases the MACC1-expressing cell portion at G2/M-Phase. However, from this assay we cannot entirely distinguish between cell cycle arrest and delay of cell cycle progression. The saffron effect on cell cycle arrest was strongly reduced in the tested CRC cell lines with low MACC1 expression (SW480/Control and SW620/KO-MACC1).

These findings establish that saffron primarily causes a MACC1-dependent cell cycle arrest. Several studies have speculated that saffron might also act through induction of apoptosis [16,39,40]. This was not the case in the MACC1 system. Indeed, protein expression analysis showed that apoptosis has remained the same with no increased caspase-3 levels after saffron exposure.

We tested different major saffron compounds in the MACC1-expressing cell system. Exposure of SW480/MACC1 and SW620/Control cells, both with high MACC1 expression, to various concentrations of crocetin showed no proliferation inhibition. Safranal reduced the proliferation rate of the CRC cells independent of the MACC1 level. This suggests a different mode of action of this compound. Indeed, Amin et al. have reported that safranal induces an ER-stress-mediated cell death accompanied by DNA double-strand breaks in hepatocellular carcinoma cells, but no cell cycle arrest [41]. Crocin was found to be the main compound that could reproduce the effects of total saffron crudes in both cell lines with MACC1 expression (SW480/MACC1 and SW620/Control) in a MACC1- and concentration-dependent manner. We have shown that the MACC1-induced proliferation and migration are reversed due to a delay/block in cell cycle progression at the G2/M phase. These results were further complemented by an independent liquid chromatography. This analysis showed that saffron 3 contained the highest crocin amount among the four saffron crudes used in our study. Saffron 3 led to the highest reduction of proliferation and migration of high MACC1-expressing CRC cells.

Several studies assigned most of the observed anti-metastatic effects of saffron to its crocin content in different types of cancers including breast, esophageal and melanoma [42,43,44]. Different molecular mechanisms of action of crocin have been described in these systems. A review by Hoshyar et al. summed up crocin´s inhibitory effects on cancer cell proliferation and induction of apoptosis through various mechanisms [45]. Thus, the here identified direct connection to MACC1 is a novel mechanism of crocin.

Rangarajan et al. suggested that saffron’s crocetinic acid can reduce DCLK1 levels in pancreatic cancer [23]. We have shown here the interaction of MACC1 with the CSC marker DCLK1. Subsequently, MACC1 gets phosphorylated by DCLK1. DCLK1 promotes tumor initiation and metastasis formation in different tumor entities including breast, CRC, and pancreas [46,47,48]. It was first described as a kinase important for motility of neurons during fetal brain development [49]. DCLK1 is a serine/threonine kinase with association to the microtubule network, where it contributes to kinesin-3-dependent cargo transport [50]. Its exact role and molecular functions are not fully understood. DCLK1 can act as an intestinal CSC marker and is a potential target for novel therapies in CRC [51]. As for MACC1, DCLK1 increases in CRC during the transition from early-stage adenoma to more advanced dysplasia [52]. Mohammadi et al. have shown the regulation of DCLK1 by miR-200. Loss of the anti-cancer miR-200c leads to an upregulation of DCLK1 and increased cellular motility [53]. This might be attributed to the higher activation of MACC1 by DCLK1 in the MACC1-positive cell lines SW620/Control and SW480/MACC1 used in this study. We have shown that saffron and crocin affect DCLK1 expression levels. DCLK1 was also reported as a target for crocetinic acid when analyzing pancreatic cancer in vitro and in vivo [23]. This saffron compound reduced the hedgehog signaling activity, affecting pancreatic CSCs, resulting in inhibited pancreatic tumorigenesis [23]. This highlights the relevance of DCLK1 as a major target in two different cancer entities (colon and pancreas), targeted by two different compounds of saffron (crocin and crocetin).

Treatment with the DCLK1 inhibitor LRRK2-IN-1 first decreased the MACC1 mRNA expression at lower inhibitor doses, but increased the MACC1 mRNA levels at higher doses. A possible explanation for the MACC1 increase during DCLK1 inhibition could be related to the reduced availability of phosphorylated, potentially activated MACC1. This might trigger the activation of MACC1 expression to substitute the missing MACC1 activity.

The kinase DCLK1 is involved in stemness and in stem cell niche formation [54]. DCLK1 is involved in numerous processes like proliferation, cell survival, drug resistance, angiogenesis, EMT, invasion, and metastasis [48,55,56,57,58,59]. All these cellular features are mediated by MACC1 too [32]. How the saffron crudes and crocin exert their effects on MACC1-dependent proliferation and migration is not known. However, it was already described that saffron can inhibit DCLK1 gene expression [22]. We identified and validated the interaction of MACC1 and DCLK1. This is, at least in part, a mechanistic basis for the cancer and metastasis inhibitory effect of saffron.

Taken together, the findings of this study reveal the MACC1-dependent anti-proliferative and anti-migration effects of saffron on CRC cell lines. In conclusion, for MACC1-driven cells, the anti-migration effect of saffron is strongly mediated by crocin. This is paralleled by a decrease in proliferation and migration via reversible G2/M cell arrest. In summary, saffron is a promising herb for the treatment of MACC1-expressing CRC. In addition, saffron might complement current standard therapy modalities like radio- and chemotherapy. The application of saffron or its main compound crocin for treatment of MACC1-driven CRC warrants further investigation.

## Supplementary Materials

The following are available online at https://www.mdpi.com/2073-4409/9/8/1829/s1, Figure S1: Supplementary Figure S1. MACC1 expression levels for the cell lines used in this study. Figure S2: Supplementary Figure S2. Saffron treatment does not reduce the proliferation rate of the MACC1 expressing cells at low Saffron concentrations. Table S1: Supplementary Table S1. Primers used for RT-qPCR.
