# Supplementary material for: Saffron Crudes and Compounds Restrict MACC1-Dependent Cell Proliferation and Migration of Colorectal Cancer Cells
Source: Cells. 2020 Aug 3;9(8):1829. doi: 10.3390/cells9081829 (PMC7463853; doi:10.3390/cells9081829)
Supplement: Supplementary file 1 [file cells-09-01829-s001.pdf]

**Supplementary Materials**

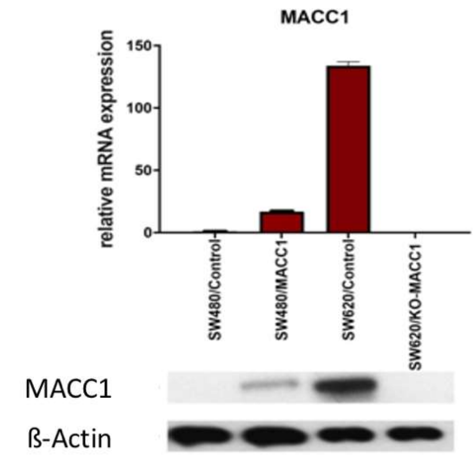


**Supplementary Figure S1.** MACC1 expression levels for the cell lines used in this study. Relative MACC1 expression levels were assessed by RT-qPCR and Western blot for SW480/Control, the MACC1 overexpressing cell line SW480/MACC1, SW620/Control and the MACC1 deficient cell line SW620/KO-MACC1.

**Supplementary Table S1.** Primers used for RT-qPCR.

| Gene | Primer | Sequence |
|---|---|---|
| MACC1 | Fwd | 5`-TTC TTT TGA TTC CTC CGG TGA-3` |
| | Rev | 5`-ACT CTG ATG GGC ATG TGC TG-3` |
| DCLK1 | Fwd | 5`-CAA CAT ACG TGG CTC CAG AA-3` |
| | Rev | 5`-CGC TGA TCT ACA TCG ACC AA-3` |
| hG6PDH | Fwd | 5`-ATC GAC CAC TAC CTG GGC AA-3` |
| | Rev | 5`-TTC TGC ATC ACG TCC CGG A-3` |

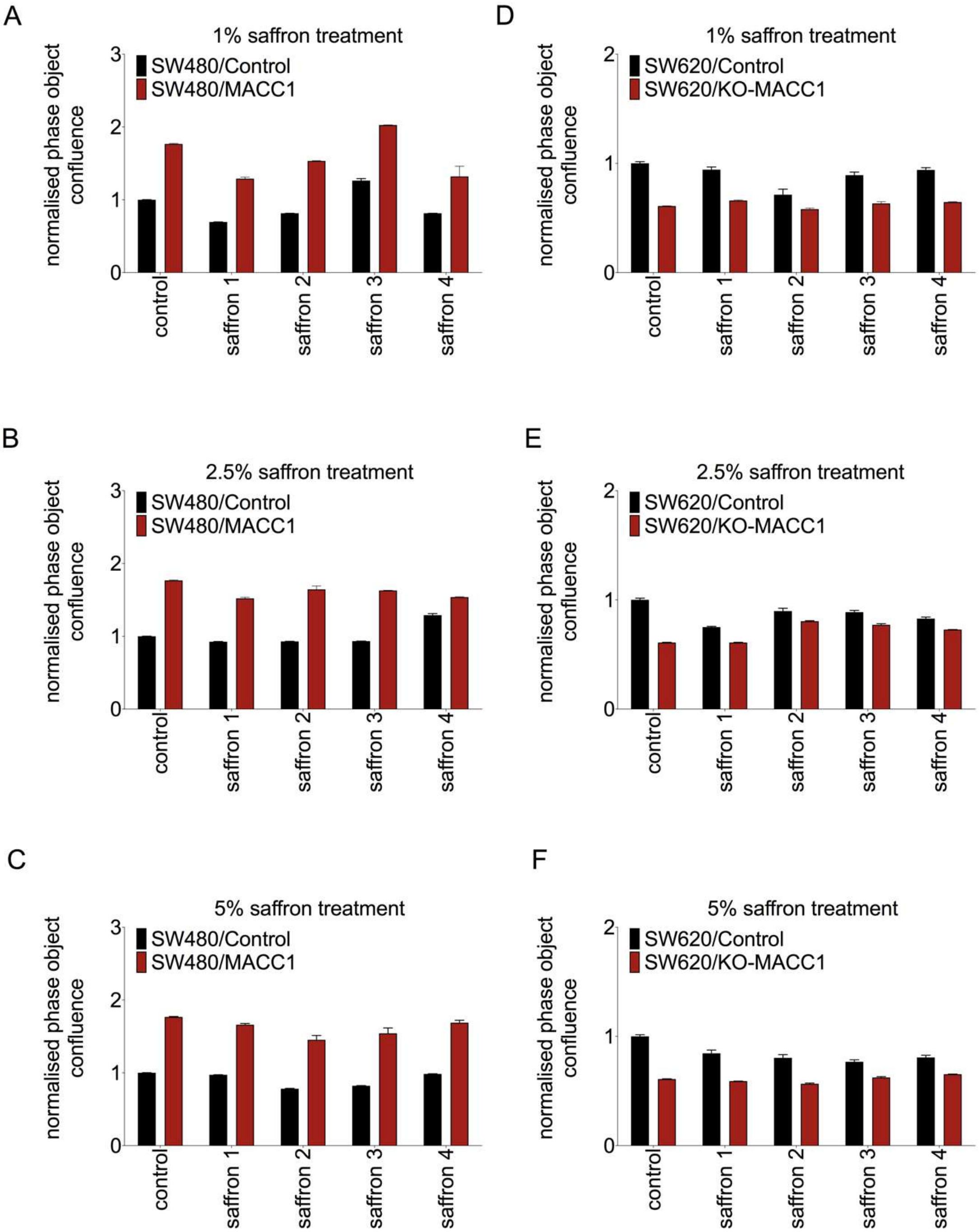


**Supplementary Figure S2.** Saffron treatment does not reduce the proliferation rate of the MACC1 expressing cells at low Saffron concentrations. CRC cells (**A**–**F**) were treated with 4 different saffron crudes with the concentrations of 1%, 2.5%, and 5% (SW480/control and SW480/MACC1 **A**–**C**, SW620/control and SW620/KO-MACC1 **D**–**F**). Independent of MACC1 level, no proliferation reduction has been observed at these low concentrations.
